# Genetics Analysis Workshop 16 Problem 2: the Framingham Heart Study data

**DOI:** 10.1186/1753-6561-3-s7-s3

**Published:** 2009-12-15

**Authors:** L Adrienne Cupples, Nancy Heard-Costa, Monica Lee, Larry D Atwood

**Affiliations:** 1 Department of Biostatistics, Boston University School of Public Health, 801 Massachusetts Avenue, Boston Massachusetts 02118, USA; 2 Framingham Heart Study, 73 Mount Wayte Avenue, Framingham, Massachusetts 01702-5728, USA; 3 Department of Neurology, Boston University School of Medicine, 75 East Newton Street, Boston Massachusetts 02118, USA

## Abstract

Genetic Analysis Workshop 16 (GAW16) Problem 2 presented data from the Framingham Heart Study (FHS), an observational, prospective study of risk factors for cardiovascular disease begun in 1948. Data have been collected in three generations of family participants in the study and the data presented for GAW16 included phenotype data from all three generations, with four examinations of data collected repeatedly for the first two generations. The trait data consisted of information on blood pressure, hypertension treatment, lipid levels, diabetes and blood glucose, smoking, alcohol consumed, weight, and coronary heart disease incidence. Additionally, genotype data obtained through a genome-wide scan (FHS SHARe) of 550,000 single-nucleotide polymorphisms from Affymetrix chips were included with the GAW16 data. The genotype data were also used for GAW16 Problem 3, where simulated phenotypes were generated using the actual FHS genotypes. These data served to provide investigators with a rich resource to study the behavior of genome-wide scans with longitudinally collected family data and to develop and apply new procedures

## Introduction

The Framingham Heart Study (FHS) -- under the direction of National Heart, Lung, and Blood Institute (NHLBI) -- began in 1948 with the recruitment of adults from the town of Framingham, Massachusetts. At the time, little was known about the general causes of heart disease and stroke, but death rates for cardiovascular disease (CVD) had been increasing steadily since the beginning of the 20^th ^century and had become an American epidemic. Even though rates of CVD have declined in recent decades, it remains the primary cause of death in both men and women in the US and in many other parts of the world. The FHS is now conducted in collaboration with Boston University.

The objective of the FHS was to identify the common factors or characteristics that contribute to CVD by following its development over a long period of time in a large group of population-based participants who had not yet developed overt symptoms of CVD or suffered a heart attack or stroke [1]. This project was unusually ambitious. As one of the first population-based, epidemiologic studies, it planned to follow participants prospectively for 20 years with repeat examinations.

The NHLBI selected Framingham, Massachusetts because it was a moderate-sized town with a relatively stable population that was thought to reflect many communities in the US at that time. In the late 1940s an estimate of the number of residents in the age range 30-60 years who were eligible for recruitment was about 10,000 individuals. The study aimed to recruit approximately 6,000 participants. Between 1948 and 1953, the researchers recruited 5,209 participants (2,336 men and 2,873 women) between the ages of 29 and 62 and began the first round of extensive physical examinations and lifestyle interviews that they would later analyze for common patterns related to CVD development. Participants were recruited from lists of addresses recorded for the town census. Recruiters approached two out of every three households for participation in the study. While there was no intention to recruit families for family studies, the plan was to recruit all household members in the ages 30-60 within each house selected for study. Hence, the Study recruited many related individuals, including siblings, parent-child dyads, and 1,644 spouse pairs. The investigators also thought that recruitment of spouse pairs would encourage continued participation for the original 20-year planned duration for the study. Since 1948, these participants have returned to the study every 2 years for a detailed medical history, physical examination, and laboratory tests. Now at more than 60 years of follow up, there remain fewer than 500 participants from this cohort, known as the Original Cohort.

Between 1971 and 1975 the Study enrolled a second-generation group -- 5,124 of the original participants' children and the spouses of these children -- to participate in similar examinations. An important goal in recruiting this Offspring Cohort was to evaluate the heritable factors involved in the development of CVD and its risk factors. Thus, the main strategy was to recruit offspring where both parents participated in the Original Cohort and those with one parent at higher risk of CVD due to higher lipid levels. Of those recruited, 2,616 participants are offspring of the original spouse pairs and 34 are stepchildren. Another 898 offspring are children of cohort members where only one parent was a study participant and 1,576 are spouses of the offspring. The Offspring Cohort participants have returned every 4 years through 2001 (except between Exams 1 and 2, which had an intervening 8 years) for follow-up exams, using protocols similar to those used for study of the Original Cohort.

Between 2002 and 2005 the Study enrolled the third generation (Generation 3) into the FHS - 4,095 offspring of the second generation. None of their spouses were recruited. At this time, the FHS also recruited an additional 103 parents of this third generation who were not recruited between 1971 and 1975. Data on the latter group are not included in the GAW16 data. With the recruitment of this third generation, the Study has increasingly focused on genetic factors associated with the development of CVD and its associated risk factors. The Generation 3 Cohort is now participating in its second examination. A description of the recruitment of this third generation and comparison with the earlier generations at their initial recruitment is presented in Splansky et al. [2].

We owe a great deal of gratitude to the Framingham participants for this rich resource of data that has accrued over 60 years in three generations. It is their unflagging commitment to the Study that makes our research possible. Further information on the Study can be found at the Framingham Heart Study web site [3].

### Genotype data

In-depth genetic studies did not begin in the FHS until the 1990s. In the late 1980s, family structures were formally formed into extended pedigrees. Also in the late 1980s and through the 1990s, investigators extracted DNA from blood samples of surviving FHS participants. Because many Original Cohort members had died by this time, study investigators obtained DNA samples from less than 30% of this cohort. In the mid-1990s into the early 2000s, the NHLBI Mammalian Genotyping Service, Center for Medical Genetics genotyped genome-wide microsatellites over several phases in the largest 330 families in the Study. And in the early 2000s, a 100 k Affymetrix genome-wide scan was conducted in these families [4]. In 2007, the FHS entered a new phase with the establishment of the FHS SHARe (SNP Health Association Resource) project by NHLBI and Boston University, for which Affymetrix performed dense SNP genotyping using approximately 550,000 SNPs (GeneChip^® ^Human Mapping 500 k Array Set and the 50 k Human Gene Focused Panel) in 10,775 samples (some duplicates) from the three generations of participants (including over 900 pedigrees). The genotyping platforms for the FHS SHARe project were the 250 k Sty, the 250 k Nsp, and the supplemental gene-centric 50 k chip. The Study obtained DNA for 89% of the participants during the 1990s. To maximize the power of the study, we also extracted DNA from 1,133 blood samples, drawn from participants who had no available DNA samples, to include in the SHARe project. These samples had been sitting in our freezers for some time, a few as far back as the 1970s. We refer to these DNA samples as the legacy samples. These samples had a higher failure rate in the genotyping process (40%) than the other 89% (3%). As a result, to maximize the number of subjects included we used different criteria for a sample to succeed in genotyping for these two types of DNA samples. All non-legacy samples must succeed on all three platforms, while legacy samples needed to pass on at least one platform. When a sample failed, additional attempts were made. Samples that repeatedly failed two to four times were called failures. Other samples failed due to issues of genotyped sex identification not matching our records, low SNP concordance among SNPs common across arrays, or contamination. Eighty-nine percent of the legacy samples for which genotyping results are available passed all three platforms. The genotyping data for the 10,043 samples from 9,354 participants that passed the Affymetrix criteria were additionally checked for sex consistency and consistency with family structure, resulting in genotyping data for 9,274 participants in FHS SHARe. Genotype calls were made with the BRLMM algorithm.

The National Center for Biotechnology Information database of Genotypes and Phenotypes [5] houses the SHARe database containing all ~550,000 SNPs and extensive phenotype data. This genome-wide dense SNP scan and a subset of phenotypes from the FHS were the focus of the Genetic Analysis Workshop 16. These same genotypes were also used to simulate the phenotype data for GAW16 Problem 3 [6]. Recently, up to ~2.5 M imputed SNPs have been added to dbGaP, but these genotypes were not available for the GAW16.

### Data for Genetic Analysis Workshop 16

The FHS data sets for Genetic Analysis Workshop 16 include pedigree, genotype, and phenotype data. The phenotypic data provide information on those participants who have consented to anyone's use, including those at for-profit and not-for-profit institutions. The pedigree file contains all biologically related participants in the FHS and is not limited to the 7,230 participants with full consent. A total of 7,130 participants have phenotype data: 373 Original Cohort, 2,760 Offspring Cohort, and 3,997 Generation 3 participants. No phenotypes were included from the 100 fully consenting non-offspring spouses. Of the 7,230 consenting participants, 6,979 are members of pedigrees and 251 are unrelated. Overall, there are a total of 6,848 participants who are genotyped, including 6,621 in pedigrees and 227 unrelated participants. There are 766 pedigrees with 2 to 301 genotyped participants: 134 pedigrees with 2, 123 with 3, 98 with 4, 85 with 5, 177 with 6 to 10, 72 with 11 to 15, 30 with 16 to 20, and 47 with more than 20.

We selected data from a subset of examinations for Genetic Analysis Workshop 16: Exams 1 (1948-1953), 4 (1954-1958), 7 (1960-1964), and 11 (1968-1971) for the Original Cohort; Exams 1 (1971-1975), 3 (1983-1987), 5 (1991-1995), and 7 (1998-2001) for the Offspring Cohort; and Exam 1 for the Generation 3 Cohort. We chose these exams so that data from FHS participants of approximately the same age from the three cohorts were considered. Only one exam had been completed for Generation 3 and so only data from one exam were available for these participants. Age, sex, and descriptive statistics for these participants are provided in Table 1. Note that Original Cohort participants with data included only the select few who survived ~40 years to have DNA collected and to provide consent for the SHARe project.

**Table 1:** Age and sex of Framingham Heart Study participants for GAW16 by cohort and exam

Variable	Original Cohort	Offspring Cohort	Generation 3 Cohort
Recruited sample size	5209	5124	4095
Number with 550 k genotype data	1529	3753	3893
Sample size for GAW16	373	2760	3997
Sample size for GAW16 with genotypes	357	2584	3811
Ages (Mean ± SD)			
Exam 1	34.9 ± 3.9	33.7 ± 9.3	40.2 ± 8.8
Exam 4 (Original), Exam 3 (Offspring)	40.9 ± 3.9	46.3 ± 9.3	NA
Exam 7 (Original), Exam 5 (Offspring)	47.0 ± 3.9	53.3 ± 9.2	NA
Exam 11 (Original), Exam 7 (Offspring)	54.7 ± 3.8	60.2 ± 9.1	NA
% Female, Exam 1	69.2%	54.4%	53.3%

Genotype data sets contained ~550,000 genotypes for each participant. We cleaned genotype data for familial relationships. We evaluated whether the genotypes of participants were consistent with their reported familial relationships. We used PREST [7] and sib-kin from Aspex [8] to perform this analysis within families [9]. Additionally, we checked for unknown (cryptic) first-degree relationships between families using PLINK [10,11]. In some cases, we altered familial relationships as a result. Such errors could occur from unknown familial relationships or sample mix-up. Cleaning at this stage could result in all genotypes of some individuals being deleted. The genotype data set included legacy DNA samples, which were of poorer quality with a higher rate of missing genotypes. Files with allele intensities and confidence scores for each marker and .cel files were also available at dbGaP [5].

The family structure file, defining the pedigree structures, was provided. This file also included indicators for which cohort a participant belongs to and whether the participant is genotyped or phenotyped. There were 8,732 participants in this file who have been genotyped. However, only data for those participants who consented to general use (both for-profit and not-for-profit) were available to GAW16.

Participants with phenotype data who are not in the family file were not members of families and were biologically unrelated to one another.

Three phenotype files are provided: 1) Original Cohort participants, 2) Offspring Cohort participants, 3) Generation 3 Cohort participants. These files provide information on demographics (sex and age), height, weight, traditional risk factors for coronary heart disease (blood pressure and hypertension, diabetes and blood glucose, smoking, alcohol, and lipid levels), and on incident coronary heart disease and age at onset. Also included are age at onset of diabetes, age at death, and age at last contact. These participants were followed up for events through 2006.

## List of abbreviations used

CVD: Cardiovascular disease; FHS: Framingham Heart Study; GAW16: Genetic Analysis Workshop 16; NHLBI: National Heart, Lung, and Blood Institute

## Competing interests

The authors declare that they have no competing interests.
